# Do Routine Gastric and Duodenal Biopsies Add Value in Patients with Eosinophilic Esophagitis? Evidence from a Large Single-Center Case Registry

**DOI:** 10.3390/diagnostics16101446

**Published:** 2026-05-09

**Authors:** Elena Grueso-Navarro, Victoria Úbeda-Vargas, Rocío Juárez-Tosina, Laura Arias-González, Emilio J. Laserna-Mendieta, Alfredo J. Lucendo

**Affiliations:** 1 Department of Gastroenterology, Hospital General de Tomelloso, 13700 Tomelloso, Spainajlucendo@sescam.jccm.es (A.J.L.); 2 Centro de Investigación Biomédica en Red de Enfermedades Hepáticas y Digestivas (CIBERehd), Instituto de Salud Carlos III, 28029 Madrid, Spain; 3 Department of Pathology, Hospital General La Mancha Centro, 13600 Alcázar de San Juan, Spain; 4 Instituto de Investigación Sanitaria Princesa, 28006 Madrid, Spain

**Keywords:** eosinophilic esophagitis, biopsy, diagnosis, guidelines, eosinophilic gastritis, prevalence, celiac disease, *Helicobacter pylori*

## Abstract

**Background/Objectives:** The recommendation of routine gastric and duodenal biopsies at index endoscopy on suspicion of eosinophilic esophagitis (EoE) remains controversial because of the limited supporting evidence. We aimed to evaluate the prevalence, clinical relevance, and predictors of gastric and duodenal histopathology in a large real-world EoE cohort. **Methods:** This retrospective single-center study included adult and pediatric patients with EoE diagnosed between 2001 and 2024 and enrolled in the EoE CONNECT registry. Gastric and/or duodenal biopsies obtained at diagnosis or follow-up were reviewed. Histologic findings were classified into specific diagnostic categories and analyzed according to clinical presentation, endoscopic features, and age group. **Results:** We evaluated 340 patients (35.7% children), of whom 88.5% underwent both gastric and duodenal biopsies. The overall diagnostic yield for extra-esophageal findings was 20.9%, predominantly *Helicobacter pylori*-associated gastritis (18.8%), but with a very low yield of clinically meaningful diagnoses such as celiac disease (0.6%) and eosinophilic gastritis (0.3%). Symptoms and endoscopic abnormalities were not significantly associated with pathological findings (p=0.11). Moreover, age-stratified analysis revealed no significant differences in specific diagnoses, although patients with pathological extra-esophageal biopsies were significantly older (p<0.001). Follow-up biopsies demonstrated resolution of most pathological findings after the initial diagnosis, with *Helicobacter pylori*-associated gastritis accounting for the majority of cases that resolved. **Conclusions:** Routine extra-esophageal biopsies in the context of patients with EoE have a low diagnostic yield and rarely alter clinical management, regardless of age. Therefore, our findings advocate for a selective rather than universal approach to extra-esophageal sampling in patients with EoE.

## 1. Introduction

Eosinophilic esophagitis (EoE) is a chronic, immune-mediated allergic disease predominantly triggered by common food antigens [1,2]. EoE impairs normal esophageal function and most commonly presents with dysphagia [3]. Histologically, EoE is characterized by dense eosinophilic infiltration of the esophageal mucosa that, by definition, does not extend beyond this organ [4]. Exclusion of alternative causes of esophageal eosinophilia, such as eosinophilic gastritis (EG) or enteritis, hyper eosinophilic syndrome, or drug hypersensitivity, among others [5], has been incorporated into the diagnostic algorithm since the first consensus document on EoE management [6].

The evolution of clinical practice guidelines and consensus documents reflects an ongoing debate regarding the utility of routine extra-esophageal biopsies. Despite being consistently recommended, this practice remains supported by a low level of evidence. Early consensus statements [6] advised universal sampling of the duodenum and stomach; however, supporting data were limited, and most available studies reported normal extra-esophageal mucosa, with only a few describing a negligible proportion of extra-esophageal findings [7,8]. In 2011, recommendations introduced distinct approaches for adult and pediatric patients, advocating a more selective strategy in adults while maintaining routine sampling in children at index endoscopy [9]. This selective approach for adults was upheld in the 2013 American College of Gastroenterology (ACG) guidelines [10], which identified abdominal pain and diarrhea as indicative symptoms for extra-esophageal sampling, although the supporting evidence was still graded as low. Subsequent evidence-based guidelines recommended gastric or duodenal biopsies without age stratification [5], whereas pediatric-focused documents provided no further directive [11,12,13]. In its 2022 consensus conference, the American Society for Gastrointestinal Endoscopy (ASGE) [14] specified that endoscopic features such as erythema, nodularity, or ulceration may suggest non-EoE eosinophilic disorders and emphasized that, in the absence of clinical features, non-esophageal mucosa is much less likely to be pathological than the esophagus [14,15]. Notably, this statement did not achieve consensus, highlighting persistent debate. The most recent ASGE consensus [16] stated that obtaining extra-esophageal biopsies at index endoscopy for children is critical. Similarly, the latest EoE guidelines maintained a strong recommendation without providing specific considerations for pediatric patients [17].

Empirical evidence addressing this issue is scarce and relatively recent. Three observational studies published between 2017 and 2022 in adult [18,19] and pediatric [20] populations consistently demonstrated a remarkably low diagnostic yield of extra-esophageal biopsies. Reported rates of extra-esophageal findings ranged from 11% to 30%; however, that included non-specific conditions such as chemical or inactive gastritis and intraepithelial lymphocytosis. Importantly, when restricting analysis to clinically meaningful diagnoses in the context of EoE, such as eosinophilic gastrointestinal diseases (EGIDs), diagnostic yield was consistently below 3% [18,19,20]. Other non-EGID conditions more frequently identified included *Helicobacter pylori* (*H. pylori*) gastritis or celiac disease (CeD), overall accounting for fewer than 5% of cases [18,19].

Although informative, these studies are limited by relatively small sample sizes [19,20], restricted age representativity [20] and incomplete assessment of the downstream clinical impact of detected abnormalities [18]. The present study leverages a large, well-characterized cohort of adult and pediatric patients to assess the clinical utility of extra-esophageal biopsies and to provide additional evidence to inform further clinical recommendations.

## 2. Materials and Methods

### 2.1. Study Design and Data Collection

We conducted a retrospective, single-center, observational study using registered data at EoE CONNECT by Hospital General de Tomelloso (Spain). EoE CONNECT, the “European Registry of Clinical, Environmental and Genetic Determinants in Eosinophilic Esophagitis”, is an international, multi-center, non-interventional registry [21] supported by the European Consortium for Eosinophilic Diseases of the Gastrointestinal Tract (EUREOS) (www.eureos.online).

We included patients diagnosed with EoE between 2001 and 2024, up to the 17th of January 2025. All included patients were required to have a histologically confirmed EoE diagnosis (≥15 eosinophils per high-power field [hpf]) and have registered at least one set of gastric or duodenal biopsies either in endoscopy at diagnosis or during follow-up visits. Patients with biopsies at follow-up were only included when no biopsy record was available at diagnosis. Records without histologic confirmation of EoE, cases lacking any gastric or duodenal biopsy at diagnosis or follow-up, and entries without pathology reports were excluded from the study. In all included patients, extra-esophageal biopsies were obtained systematically per EoE clinical practice guideline recommendations, irrespective of whether sampling occurred at the index endoscopy in our hospital or at a subsequent endoscopy in patients referred without prior (or without documented) gastric or duodenal biopsies.

The hospital database was searched for corresponding endoscopy and pathology reports, retrieved from the available digital clinical records. From endoscopy and pathology records, the following variables were extracted: indication for endoscopy, endoscopic diagnosis, biopsy site (duodenum or stomach), peak of eosinophil count per biopsy set, and histopathological diagnosis. EoE CONNECT provided data on patient’s sex, birth date, atopic conditions, symptoms at diagnosis and endoscopic features according to EREFS (edema, rings, exudates, furrows and strictures [22]). Only symptoms that affected at least 10% of the studied patients were reported.

For each biopsy site, three categorical variables were defined: (i) biopsy availability (yes/no); (ii) histopathological status, defined as four mutually exclusive categories: *pathological* (a specific lesion was identified in the index pathology report), *non-pathological* (no abnormal finding reported), *finally non-pathological* (the index report described non-specific changes such as duodenitis or unspecific intraepithelial lymphocytosis, and subsequent clinical, serological and/or histological follow-up did not confirm a specific diagnosis), and *not evaluable* (insufficient or non-representative material precluding diagnosis); and (iii) specific diagnostic category. Gastric categories included *H. pylori*-associated gastritis, chronic gastritis with intestinal metaplasia, fundic gland polyps, and EG (>30 eosinophils/hpf). Non-active gastritis was considered non-pathological. Duodenal categories comprised duodenitis, unspecific intraepithelial lymphocytosis, and CeD. Duodenitis and unspecific intraepithelial lymphocytosis were reclassified as *finally non-pathological* when subsequent serological testing and/or repeat duodenal biopsies excluded CeD or other specific enteropathies. Specimens not evaluable for diagnosis were not assigned a definitive diagnosis. A flow diagram summarizing the classification scheme is provided in the Supplementary Material (Figure S1).

All endoscopic procedures were performed under propofol sedation by a certified gastroenterologist with GIF-Q165 and H185 gastroscopes (Olympus Medical Systems, Hamburg, Germany). Biopsy specimens were obtained with standard needle biopsy forceps (Endo Jaw FB-220U, Olympus Medical Systems; Hamburg, Germany) and fixed in 4% formalin for further histological analysis by expert pathologists.

The study was conducted in accordance with the Declaration of Helsinki and was approved by the Comité Ético de Investigación Clínica del Hospital La Mancha Centro (protocol code 16/2015, date of approval 16 November 2015). Informed consent was obtained from all enrolled patients or their legal guardians involved in the study.

### 2.2. Statistical Analysis

In this study, categorical variables are summarized as absolute counts and percentages, whereas continuous variables are expressed as mean ± standard deviation (SD) when normally distributed or as median with interquartile range (IQR) when non-normally distributed. Data normality was evaluated with the Shapiro–Wilk test. Categorical variables were compared using the chi-square test, the Mann–Whitney U test was used for comparisons of non-normally distributed continuous variables, and the *t*-test was used for those showing normal distribution. Statistical significance was reached at p<0.05. No data imputation was applied for missing values. Statistical analysis was performed using the IBM SPSS software package v.25 (SPSS Inc., Chicago, IL, USA).

## 3. Results

### 3.1. Patient Characteristics

As of 17 January 2025, EoE CONNECT had registered 416 patient records from our hospital, of which 82.7% had at least one extra-esophageal biopsy. The histopathology reports were not available for four patients, as they were diagnosed at another site. Consequently, 340 patients were evaluated, and key demographic and clinical characteristics are summarized in Table 1.

More patients had their first extra-esophageal biopsy obtained at diagnostic endoscopy (n=192, 56.5%) than during follow-up (n=148, 43.5%) (p=0.017). To assess whether the two groups differed in baseline characteristics, disease severity, biopsy availability and diagnostic yield, we performed a comprehensive comparison summarized in Table S1. Importantly, the diagnostic yield of pathological extra-esophageal biopsies did not differ between the two groups.

### 3.2. Diagnostic Yield of Duodenal and Gastric Biopsies

**Figure 1:**
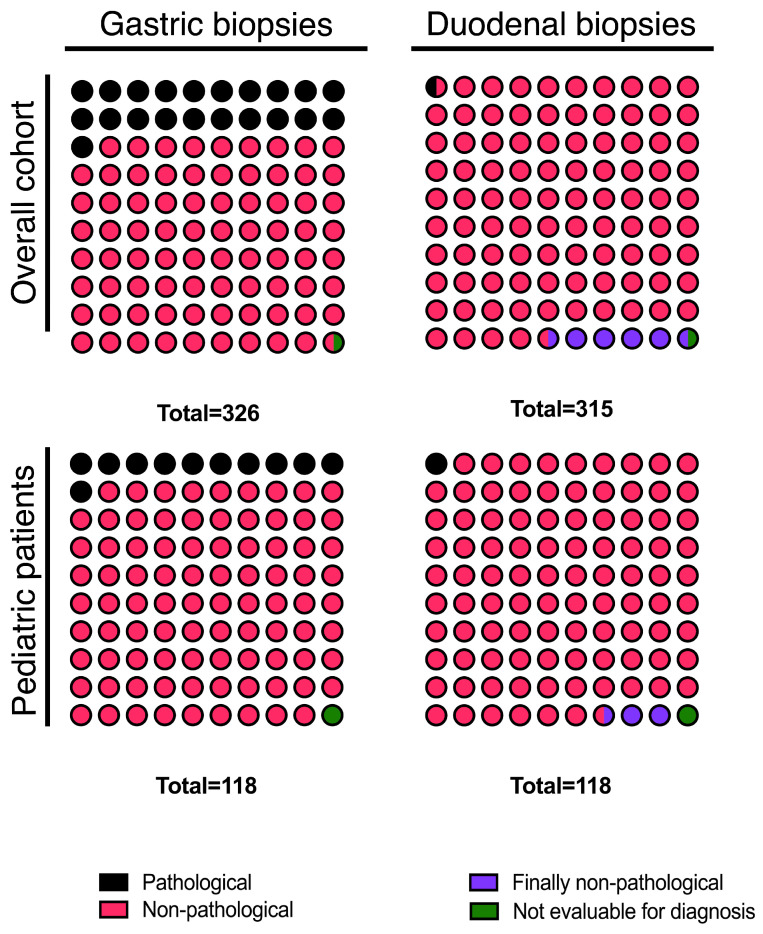
Distribution of pathological extra-esophageal biopsies in pediatric patients and the overall cohort. The total number of biopsies displayed in each panel, therefore, reflects the number of patients in whom that biopsy was performed: in the overall cohort, 326 patients had a gastric biopsy and 315 had a duodenal biopsy; in the pediatric subgroup, 118 children (97.5% of the 121 pediatric patients) underwent both gastric and duodenal sampling.

Gastric biopsies were obtained in 326/340 (95.9%) EoE patients and duodenal biopsies in 315/340 (92.6%) patients. Overall, 88.5% of EoE patients underwent both gastric and duodenal biopsies, either at index endoscopy or during follow-up. Reasons for missing biopsies were not documented in the endoscopy reports. Most samples were classified as non-pathological (gastric biopsies: 78.8%; duodenal biopsies: 94%) (Figure 1). Three biopsies were deemed “not evaluable for diagnosis” because of poor tissue representativeness or insufficient specimens.

Among gastric biopsies, 69/326 (21.2%) were classified as pathological and grouped into five pathological groups. *H. pylori* gastritis accounted for the vast majority (64/69). In total, 17 duodenal biopsies were initially reported to be pathological; however, after clinical follow-up, only two duodenal biopsies were considered pathological with a diagnosis of CeD (Table 2). Overall, the proportion of patients with pathological extra-esophageal biopsies was 20.9% (71/340), where *H. pylori*-associated gastritis accounted for 18.8% and isolated intestinal metaplasia for 0.9%, followed by CeD (0.6%). EG and fundic gland polyps were found in one patient each (0.3%).

We next evaluated whether patients with pathological extra-esophageal biopsies (n=71) differed from those without pathological findings (Table 3). These patients were significantly older at diagnosis (p<0.001), and consequently, a lower proportion of pediatric patients was observed (p=0.001). No differences were observed in symptoms reported at EoE diagnosis between groups.

We also assessed whether non-esophageal endoscopic findings were reported. A total of 321/340 patients (94.4%) had available endoscopic reports, of which 83 (25.8%) documented endoscopic abnormalities (79.5% only in the stomach, 6% only in the duodenum and 14.5% in both the stomach and duodenum) (Table S2). However, only 22/83 patients (26.5%) ultimately had pathological biopsies, and 43/238 (18.1%) of patients without reported extra-esophageal endoscopic abnormalities showed pathological extra-esophageal biopsies. Consequently, there was no association between endoscopic abnormalities and pathological findings (p=0.11).

### 3.3. Comparative Impact of Extra-Esophageal Biopsies in Pediatric Patients

We evaluated biopsy abnormalities specifically within the pediatric subgroup (n=121). Nearly all pediatric patients (97.5%) underwent gastric and duodenal sampling. Pathological gastric biopsies were more frequent than pathological duodenal biopsies, although the vast majority were non-pathological (Figure 1). *H. pylori* infection was the most common gastric finding (84.6%), whereas other conditions were absent or only rarely found in this subgroup (Table 2). We found no significant differences between adult and pediatric patients with regard to *H. pylori* infection (92.7% vs. 84.6%; p=0.33), and other conditions were exclusively observed in one or another cohort, with a very low prevalence overall (Table 2). In absolute terms, only one pediatric patient (0.8%) had a clinically meaningful alternative diagnosis (CeD), and one (0.8%) was diagnosed with EG; all remaining pathological pediatric biopsies corresponded to *H. pylori* gastritis (n=11, 9%) or isolated intestinal metaplasia (n=1, 0.8%). The corresponding figures in the adult cohort (n=219) were: CeD, 1 (0.5%); EG, 0 (0%); *H. pylori*-associated gastritis (with or without intestinal metaplasia), 53 (24.2%); isolated chronic gastritis with intestinal metaplasia, 2 (0.9%); and fundic gland polyps, 1 (0.5%).

### 3.4. Follow-Up of Pathological Extra-Esophageal Biopsies

**Figure 2:**
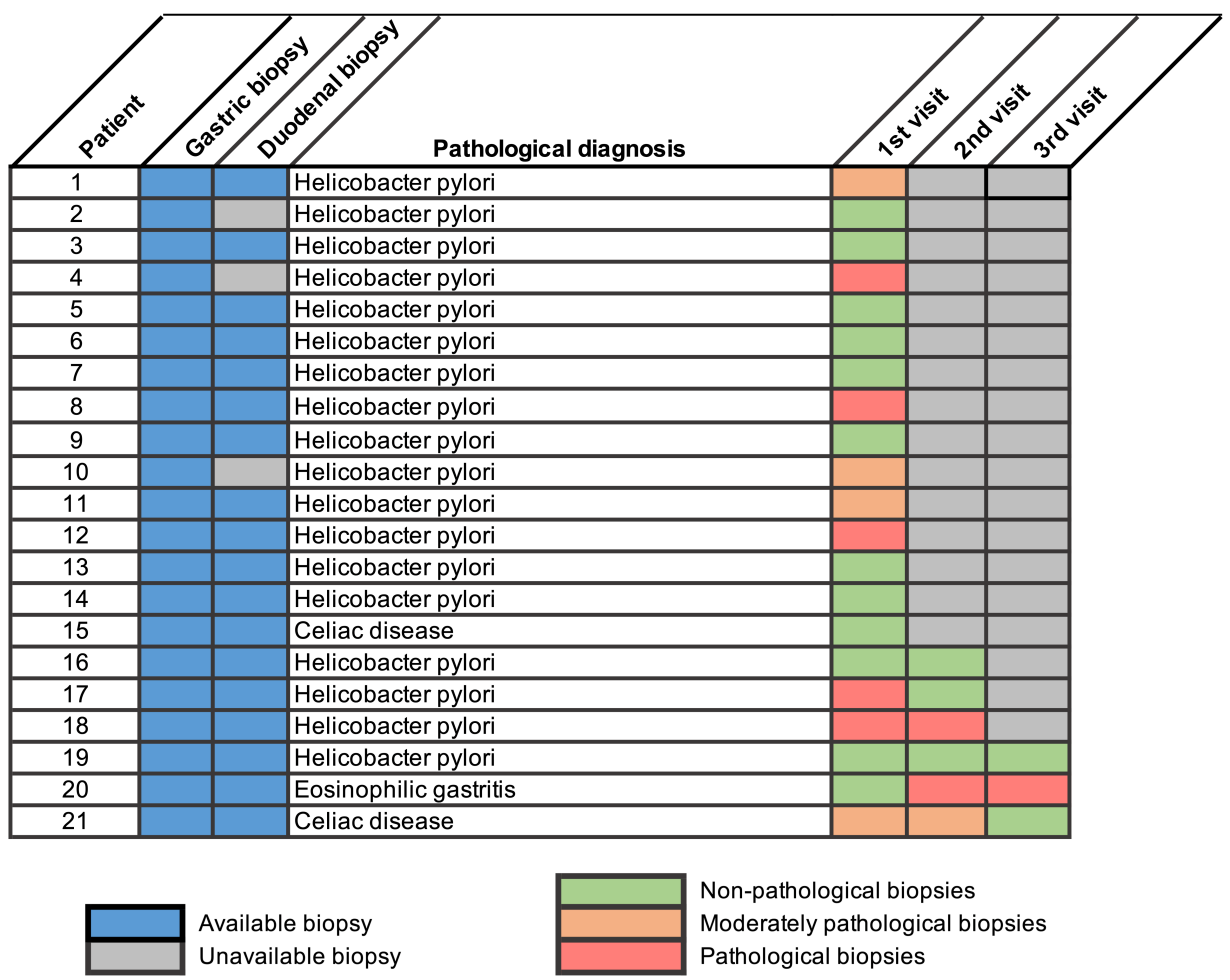
Subsequent follow-up of pathological findings in extra-esophageal biopsies. Classification as “moderately pathological” was applied to diagnosis of intestinal metaplasia (in patients 1, 10 and 11), or “reactive changes in duodenal mucosa” (in patient 21). Classification as pathological was applied in the cases when the finding at diagnosis was not resolved.

Lastly, we examined whether patients with pathological findings underwent follow-up endoscopies with repeated sampling. Among patients with extra-esophageal findings (n=71), 21 had a documented follow-up visit including extra-esophageal biopsies (29.6%), of whom 28.6% and 14.3% had second and third follow-up visits. Overall, 66.6% of patients had a resolved pathology in the first follow-up visit. For those whose latest biopsy showed *H. pylori* infection, subsequent non-invasive tests confirmed eradication of infection. Regarding the patient diagnosed with EG, the first biopsy showed no active gastric inflammation; however, the subsequent two did, coinciding with the withdrawal of oral corticosteroid treatment (Figure 2). One of the two patients who were diagnosed with concomitant CeD exhibited recurrent pathological findings at the second endoscopy, attributed to poor adherence to the gluten-free diet, assessed by the attending gastroenterologist at the follow-up visit.

## 4. Discussion

Here, we provide a comprehensive assessment of the prevalence and clinical relevance of extra-esophageal biopsies in a large, single-center cohort of adult and pediatric patients with EoE. We found that pathological findings were largely attributable to *H. pylori*-associated gastritis, whereas other clinically relevant conditions such as EG or CeD were rare. Because this condition does not alter the diagnostic criteria or therapeutic management of EoE, *H. pylori* gastritis should be regarded as an incidental finding rather than a clinically actionable result of routine extra-esophageal sampling in this setting. In univariate analyses, no symptom profile was significantly associated with the presence of pathological extra-esophageal findings (Table 3). Collectively, these findings indicate that routine extra-esophageal biopsies in EoE add limited clinical value, regardless of patient age.

Despite current recommendations supporting extra-esophageal sampling [17], both our findings and previously published data [18,19,20] indicate that symptoms and endoscopic appearances show limited diagnostic utility for identifying histologic abnormalities in most cases. Consistently, the proportion of patients with endoscopic abnormalities who had pathological biopsies was similar to that of patients without such abnormalities (18.1% vs. 26.5%; p=0.11). Nonetheless, it should be considered that, unlike EoE, where the EREFS score provides a validated framework, endoscopic findings in non-EoE EGIDs such as EG or eosinophilic enteritis exhibit normal-appearing mucosa, as reported in up to 60–70% of cases [23]. This may contribute to the under-recognition of subtle pathological signs during EoE-focused endoscopies. In addition, de Rooij et al. reported that 67% of their cohort would have qualified for extra-esophageal sampling, whereas only 1.6% were ultimately diagnosed with extra-esophageal disease, predominantly chronic gastritis, an entity with limited relevance for EoE diagnosis or management [19]. In contrast, Hiramoto et al. observed a higher prevalence of abdominal pain, dyspepsia, and endoscopic abnormalities among EoE patients with concomitant extra-esophageal findings compared with those with isolated EoE (98.6% vs. 55.6%) [18]. Taken together, these discrepancies highlight the need for studies involving larger sets of patients with systematic collection of symptoms and endoscopic data to better define their predictive value.

Our findings did not reveal significant differences in the prevalence of extra-esophageal disease between pediatric and adult patients that would justify distinct biopsy strategies based on age. Prior consensus statements [9,16] and clinical guidelines [10,17] differentiated pediatric and adult patients regarding recommendations about routine acquisition of extra-esophageal biopsies at diagnosis. Although the symptom profile of EoE in children is more heterogeneous and non-specific, with vomiting and abdominal pain being predominant symptoms [24,25], and may overlap with alternative diagnoses such as EG or CeD, epidemiological data do not support a higher prevalence of EG in children, as this condition appears to be more common in adults [26]. While CeD is indeed more prevalent in pediatric populations [27], its diagnosis in children may rely on a no-biopsy serological approach [28], whereas in adults, duodenal biopsies are still required [29]. Given that endoscopic biopsy sampling prolongs procedures and carries inherent, albeit rare, risks [16], our data support a selective rather than universal approach to extra-esophageal biopsy sampling, also in pediatric patients.

A detailed examination of clinical outcomes further questions the value of non-targeted sampling. In our cohort, the two cases of CeD prompted initiation of a gluten-free diet, representing a meaningful clinical impact. However, one of these patients already exhibited endoscopic abnormalities that would have warranted targeted biopsies irrespective of an EoE-specific protocol. When compared with the pediatric cohort reported by Kaur et al., in which 100% of CeD cases occurred in patients with a previously known history of the disease [20], the rationale for routine sampling is further weakened. Regarding EG, findings from both studies suggest that diagnoses are typically preceded by clinical or endoscopic markers or do not substantially alter management, in part because pharmacological treatments for EoE and EG largely overlap [30]. In our cohort, the single case of EG occurred in the context of suspected hypereosinophilic syndrome. Similarly, Kaur et al. reported that their EG cases (1.9%, 2/160) presented with overt endoscopic abnormalities, including pyloric and duodenal ulcers and a granular mucosal appearance [20].

In our study, we examined follow-up extra-esophageal biopsies in patients with pathological extra-esophageal findings to assess persistence of abnormalities over time. To our knowledge, evidence addressing this question is limited to a single study by Kaur et al. [20]. Consistent with our findings, they reported that only a minority of patients had persistent pathological findings after one follow-up endoscopy (35% gastric and 23% duodenal abnormal biopsies), largely restricted to inactive/chemical gastritis or CeD. In our cohort, 21 out of 71 patients (29.6%) with pathological findings at diagnosis underwent extra-esophageal biopsies during one or more follow-up endoscopies. Although follow-up sampling was performed in a relatively low subset of patients, our results align with existing evidence and further support the limited persistence and clinical relevance of extra-esophageal conditions over time.

We must acknowledge several limitations of the present study. First, our findings illustrate the utility of extra-esophageal biopsies for identifying concomitant gastrointestinal diseases rather than their role in the initial differential diagnosis or exclusion of EoE, as our data was derived from a dedicated EoE registry comprising patients with an established diagnosis. Second, as a single-center study, the generalizability of our results is limited, since the cohort largely represents a single ethnic background and may not capture geographic variability in the prevalence of other gastrointestinal conditions. Moreover, we should acknowledge the exploratory nature of our data due to the limited number of events for certain diagnoses. In addition, our institution is a referral center for EoE management; therefore, adherence to biopsy protocols and standardized clinical care may be higher than in other care settings, particularly regarding the prevalence of extra-esophageal biopsy sampling at diagnosis. Finally, the retrospective design of the study, as in similar precedent studies [18,19,20], is inherently limited by the absence of a prospective biopsy protocol applied uniformly across all patients.

Conversely, our study has several notable strengths. The use of the European EoE CONNECT registry ensured uniform and standardized data collection. This systematic approach substantially improved, compared with previous studies, the characterization of symptoms at the time of biopsy sampling, allowing us to robustly confirm the lack of association between clinical presentation and pathological gastric or duodenal biopsies. Notably, the single-center setting also enabled a comprehensive review of clinical records available within the institution, further strengthening the completeness and reliability of the clinical data analyzed.

## 5. Conclusions

In a large cohort of patients with EoE, routine gastric and duodenal biopsies—whether obtained at index endoscopy or during follow-up—had a low diagnostic yield and rarely influenced clinical management. Clinically relevant alternative diagnoses such as EG or CeD were exceptional. Neither symptom profile nor extra-esophageal endoscopic abnormalities were associated with pathological histology, and age-stratified analyses did not support a distinct benefit of routine sampling in pediatric patients. Altogether, these findings question the utility of routine extra-esophageal biopsies in the EoE diagnostic algorithm.

## Figures and Tables

**Table 1:** Clinical and demographic characteristics of evaluated patients. Unless otherwise specified, n=340 for all variables.

Variable	Value
Age at diagnosis, years (mean ± SD), range (n=338)	26.7 ± 14.9 (2–62)
<18 years old, *n* (%) (n=338)	121 (35.7)
Male sex, *n* (%)	266 (78.2)
**Number of concomitant atopic conditions, *n* (%)**	
At least 1	252 (74.1)
At least 2	200 (58.8)
At least 3	123 (36.2)
**Concomitant atopic conditions, *n* (%)**	
Rhinitis	209 (61.5)
Conjunctivitis	179 (52.6)
Asthma	131 (38.5)
Food allergies	112 (32.9)
Atopic dermatitis	60 (17.6)
Urticaria	30 (8.8)
Chronic rhinosinusitis with nasal polyps	13 (3.8)
**Clinical presentation at diagnosis, *n* (%)**	
Dysphagia	222 (65.3)
Food bolus impaction	206 (60.6)
Choking	83 (24.4)
Vomiting	71 (20.9)
Slow pace of eating	68 (20.0)
Heartburn	57 (16.8)
Failure to thrive	53 (15.6)
Abdominal pain	40 (11.8)
Chest pain	38 (11.2)
Epigastric pain	34 (10.0)

**Table 2:** Pathological findings in extra-esophageal biopsies of the overall and pediatric cohorts.

	Overall Cohort (n=340)		Pediatric Cohort (n=121)	
	n	**(%)**	n	**(%)**
**Gastric biopsies**				
*H. pylori*-associated gastritis	60	(86.9)	11	(84.6)
*H. pylori* gastritis with intestinal metaplasia	4	(5.8)	0	(0)
Chronic gastritis with intestinal metaplasia	3	(4.3)	1	(7.7)
Fundic gland polyps	1	(1.5)	0	(0)
Eosinophilic gastritis	1	(1.5)	1	(7.7)
**Duodenal biopsies**				
Celiac disease	2	(11.8)	1	(100)
Duodenitis	5	(29.4)	—	—
Intraepithelial lymphocytosis	10	(58.8)	—	—

Percentages are calculated over the total number of pathological biopsies per site and cohort (overall gastric: n=69; overall duodenal: n=17; pediatric gastric: n=13; pediatric duodenal: n=1).

**Table 3:** Comparing clinical and demographic characteristics of patients with pathological vs. non-pathological extra-esophageal biopsies.

	Pathological Biopsy (n=71)	Non-Pathological Biopsy (n=269)	*p*-Value
Male, *n* (%)	62 (89.8)	180 (76.6)	0.288
Age at diagnosis, mean (SD)	32.2 ± 15.1	25.2 ± 14.4	<0.001
<18 years old, *n* (%)	15 (21.7)	99 (42.3)	0.001
**Clinical presentation at diagnosis, *n* (%)**			
Dysphagia	48 (65.8)	151 (64.3)	0.694
Food bolus impaction	48 (65.8)	137 (58.3)	0.433
Choking	17 (23.3)	54 (23.0)	0.194
Slow pace of eating	15 (20.5)	46 (19.6)	0.946
Vomiting	13 (17.8)	50 (21.3)	0.681
Heartburn	13 (17.8)	38 (16.2)	0.902
Failure to thrive	11 (15.1)	34 (14.5)	0.302
Abdominal pain	4 (5.5)	33 (14.0)	0.127
Chest pain	8 (11.0)	28 (11.9)	0.633

## Data Availability

The data presented in this study are available on request from the corresponding author. The data are not publicly available due to patient privacy restrictions.

## Supplementary Materials

The following supporting information can be downloaded at: https://www.mdpi.com/article/10.3390/diagnostics16101446/s1, Table S1: Comparison of demographic, clinical, endoscopic and histopathological characteristics between patients whose first extra-esophageal biopsy was obtained at the diagnostic endoscopy versus during follow-up; Table S2: Extra-esophageal endoscopic abnormalities and their association with pathological biopsies; Figure S1: Flow diagram of the histopathological classification scheme.
