# Supplementary material for: Do Routine Gastric and Duodenal Biopsies Add Value in Patients with Eosinophilic Esophagitis? Evidence from a Large Single-Center Case Registry
Source: Diagnostics (Basel). 2026 May 9;16(10):1446. doi: 10.3390/diagnostics16101446 (PMC13205429; doi:10.3390/diagnostics16101446)
Supplement: Supplementary file 1 [file diagnostics-16-01446-s001.zip › Supplementary material_revised2.pdf]

Supplementary material

Table S1. Comparison of demographic, clinical, endoscopic and histopathological characteristics between patients whose first extra-esophageal biopsy was obtained at the diagnostic endoscopy versus during follow-up.

Variable	N with data	Biopsy at diagnosis (n=192)	Biopsy at follow-up (n=148)	*p-value*
Demographics				
Age at diagnosis, year (median, IQR)	338	25 (12-38)	26 (15-39)	0.466
Pediatric (< 18 years old), n (%)	340	69 (35.9)	52 (35.1)	0.969
Male sex, n (%)	340	151 (78.6)	115 (77.7)	0.939
Concomitant atopies, n(%)				
Rhinitis	314	109 (63.0)	90 (63.8)	0.974
Conjunctivitis	310	94 (54.7)	74 (53.6)	0.947
Asthma	310	68 (39.5)	65 (47.1)	0.222
Food allergies	340	60 (31.2)	52 (35.1)	0.523
Atopic dermatitis	312	25 (14.5)	37 (26.6)	**0.011**
Urticaria	300	16 (9.6)	15 (11.2)	0.803
Chronic rhinosinusitis with nasal polyps	299	2 (1.2)	2 (1.5)	—
Clinical presentation at diagnosis, n (%)				
Dysphagia	340	119 (62.0)	103 (69.6)	0.178
Food bolus impaction	340	106 (55.2)	100 (67.6)	**0.028**
Choking	340	38 (19.8)	45 (30.4)	**0.033**
Vomiting	340	40 (20.8)	31 (20.9)	1.000
Slow eating	340	40 (20.8)	28 (18.9)	0.764
Chest burn (heartburn)	340	34 (17.7)	23 (15.5)	0.701
Failure to thrive	340	29 (15.1)	24 (16.2)	0.897
Abdominal pain	340	24 (12.5)	16 (10.8)	0.757
Chest pain	340	23 (12.0)	15 (10.1)	0.718
Epigastric pain	340	20 (10.4)	14 (9.5)	0.913

Endoscopic and histologic features				
EREFS score, mean (SD)	277	3.2 (1.7)	3.2 (1.7)	0.82
EREFS inflammatory subscore, mean (SD)	233	2.4 (1.3)	2.2 (1.3)	0.19
EREFS fibrotic subscore, mean (SD)	233	0.9 (1.1)	1.3 (1.3)	0.12
Epithelial peak eosinophil count, cells/HPF, median (IQR)	333	45 (26-80)	40 (5-70)	0.005
Edema, n (%)	234	104 (63.4)	35 (50.0)	0.06
Rings presence, n (%)	275	88 (50.0)	71 (71.7)	0.001
Rings severity, mean (SD)<br>*Median (IQR)*	275	0.8 (0.99)<br>0 (0-3)	1.2 (1.1)<br>1 (0-3)	<0.001
Exudates presence, n (%)	248	115 (68.8)	47 (58.0)	0.117
Exudates severity, mean (SD)<br>*Median (IQR)*	248	0.9 (0.7)<br>1 (0-2)	0.7 (0.7)<br>1 (0-2)	0.099
Furrows, n (%)	254	135 (78.9)	67 (80.7)	0.87
Strictures, n (%)	239	25 (15.2)	28 (37.3)	<0.001
Minor findings-fragility, n (%)	233	34 (21.0)	15 (21.1)	>0.99
Extra-esophageal biopsy availability, n (%)				
Gastric biopsy obtained	340	188 (97.9)	138 (93.2)	0.061
Duodenal biopsy obtained	340	172 (89.6)	143 (96.6)	**0.024**
Pathological yield among biopsied patients, n (%)				
Pathological gastric biopsy	326	42 (22.3)	27 (19.6)	0.639
Pathological duodenal biopsy (after follow-up review)	315	2 (1.2)	0 (0.0)	—
Specific extra-esophageal diagnoses, n (%) of full group				
Helicobacter pylori-associated gastritis (any)	340	41 (21.4)	23 (15.5)	0.223
Isolated chronic gastritis with intestinal metaplasia	340	0 (0.0)	3 (2.0)	—
Fundic gland polyps	340	3 (1.6)	0 (0.0)	—
Eosinophilic gastritis	340	0 (0.0)	1 (0.7)	—

Celiac disease	340	2 (1.0)	0 (0.0)	—
Duodenitis (initial report)	340	5 (2.6)	0 (0.0)	—
Intraepithelial lymphocytosis (initial report)	340	6 (3.1)	4 (2.7)	—
Overall pathological yield				
ANY pathological extra-esophageal biopsy	340	44 (22.9)	27 (18.2)	0.359

For categorical variables in which fewer than 5 events were observed in either group, only descriptive comparisons are reported (n, %). Only symptoms with a prevalence ≥10% in the overall cohort are presented. Specific extra-esophageal diagnoses are reported with the full group N as denominator.

Table S2. Extra-esophageal endoscopic abnormalities and their association with pathological biopsies.

	**Pathologic finding**	**N***
Stomach (n=78 patients)	Patchy redness	37
	Erythematous mucosa	16
	Small polyps	7
	Antral nodularity	6
	Cardial incompetence or hiatal hernia	9
	Other findings	19
Duodenum (n=17 patients)	Villous blunting	2
	Bulbar erythema	10
	Duodenal erosions or ulcers	5
	Duodenal lymphangiectasia	2
	Deformity or pseudodiverticulum	2

*Different findings may coexist in the same patient. In total 83 patients had endoscopic findings: 66 patients had endoscopic findings in the stomach only, 5 patients had endoscopic findings in duodenum only, and 12 patients had endoscopic findings in both stomach and duodenum.
