# Supplementary figures and images for: Do Routine Gastric and Duodenal Biopsies Add Value in Patients with Eosinophilic Esophagitis? Evidence from a Large Single-Center Case Registry
Source: Diagnostics (Basel). 2026 May 9;16(10):1446. doi: 10.3390/diagnostics16101446 (PMC13205429; doi:10.3390/diagnostics16101446)

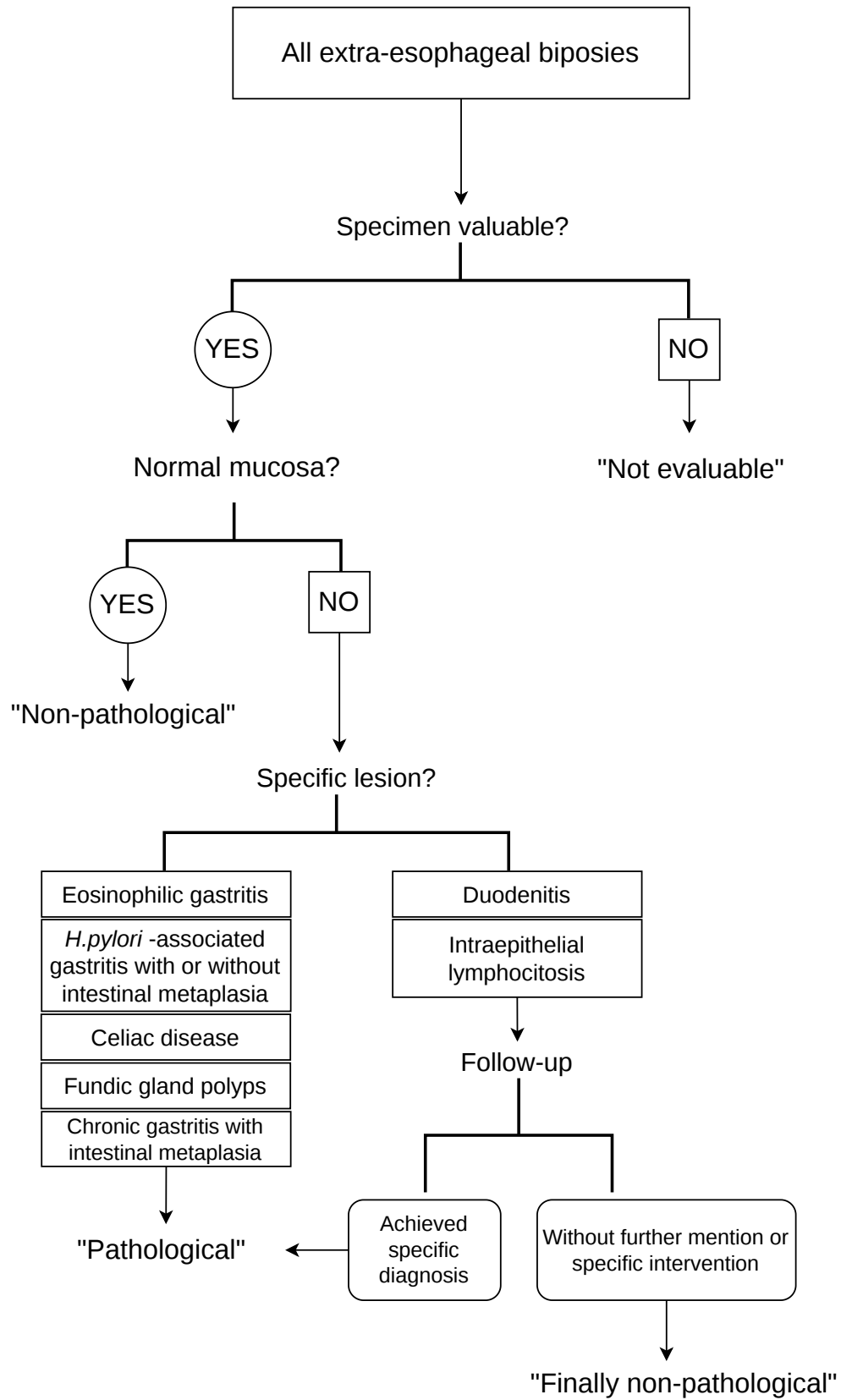

Supplement: Supplementary file 1 [file diagnostics-16-01446-s001.zip › Figure S1.drawio (2).pdf]
